# Supplementary figures and images for: Developing an enhanced 7-color multiplex IHC protocol to dissect immune infiltration in human cancers
Source: PLoS One. 2021 Feb 17;16(2):e0247238. doi: 10.1371/journal.pone.0247238 (PMC7888634; doi:10.1371/journal.pone.0247238)

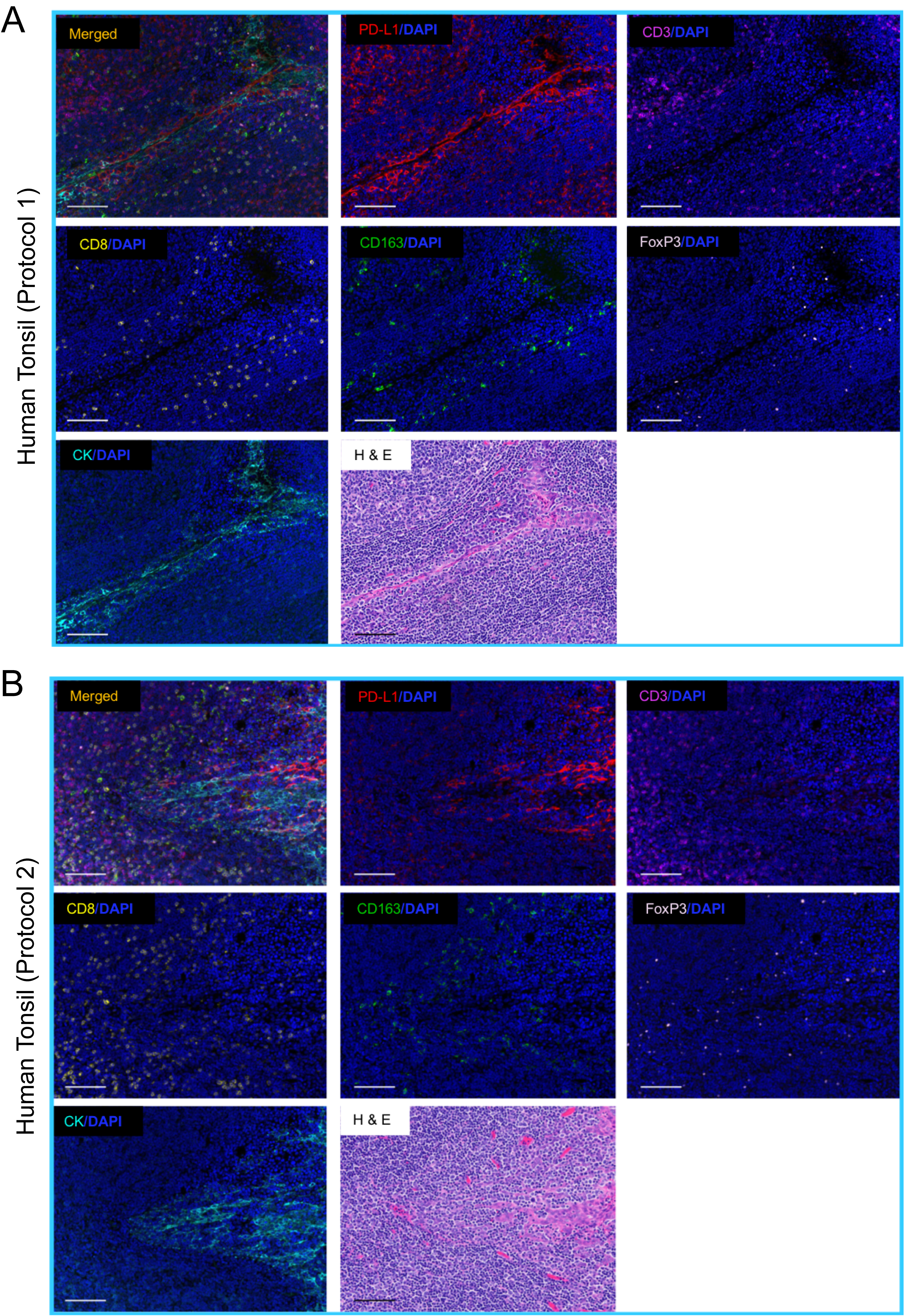

Supplement: S1 Fig — (TIF) [file pone.0247238.s001.tif]

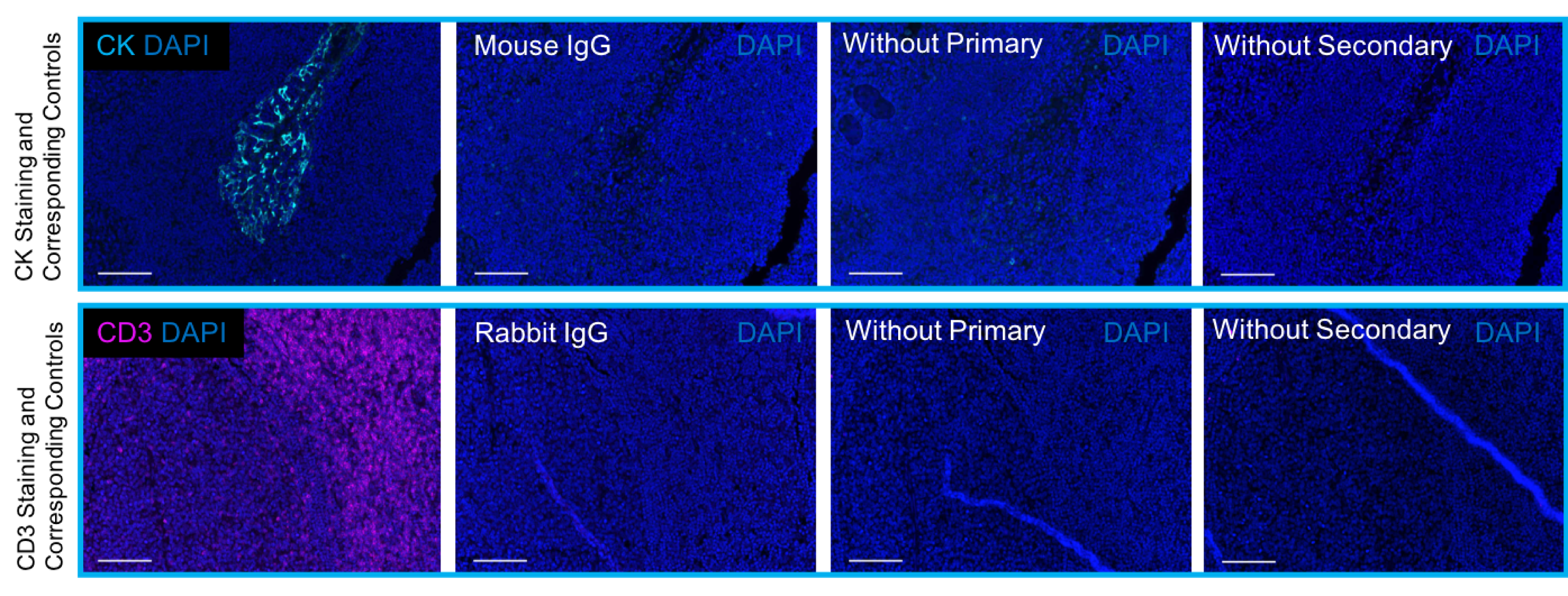

Supplement: S2 Fig — (TIF) [file pone.0247238.s002.tif]

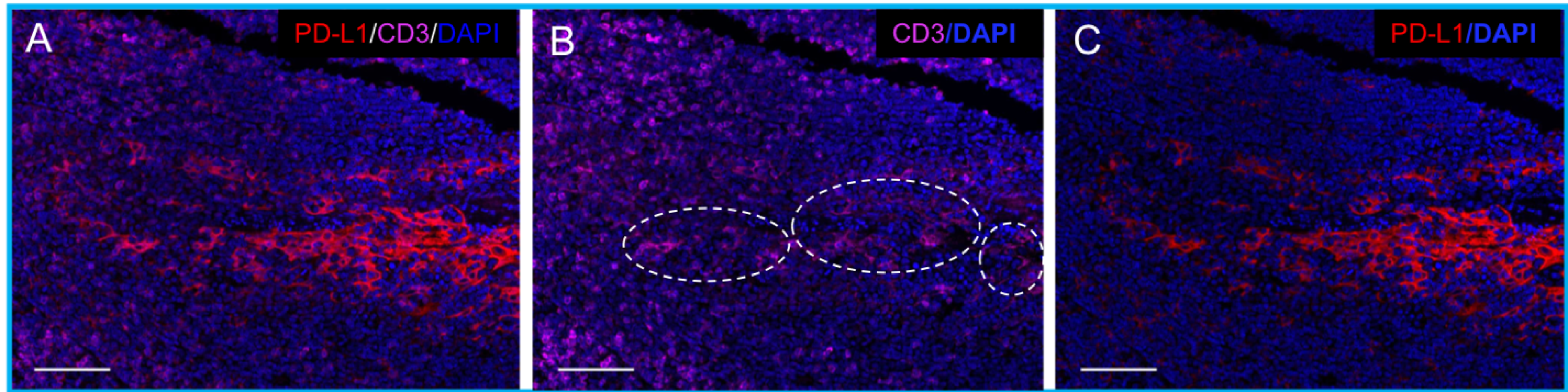

Supplement: S3 Fig — The circled cells are PD-L1+ CD3- cells. (TIF) [file pone.0247238.s003.tif]

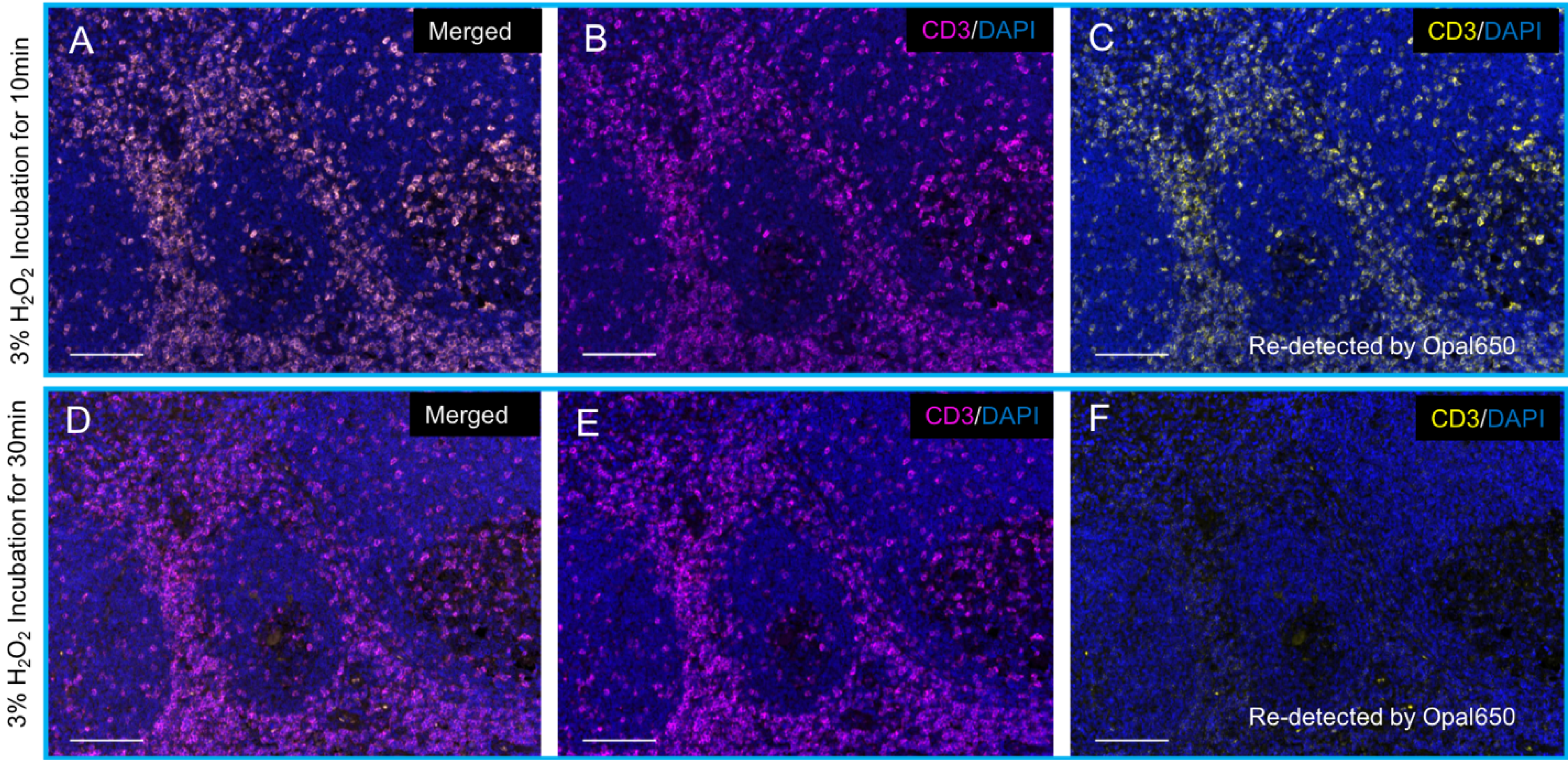

Supplement: S4 Fig — Incubation with 3% H2O2 for 30 min at RT was sufficient to inactivate exogenous HRP caused by CD3 stain (compare S3F vs. S3C). (TIF) [file pone.0247238.s004.tif]
